# Supplementary material for: Pseudomonas response regulators produced in an E. coli heterologous expression host exhibit host-derived post-translational phosphorylation
Source: Sci Rep. 2022 Jun 20;12:10336. doi: 10.1038/s41598-022-13525-2 (PMC9209504; doi:10.1038/s41598-022-13525-2)
Supplement: Supplementary file 1 — Supplementary Information 1. [file 41598_2022_13525_MOESM1_ESM.pdf]

Supplementary Materials for

Pseudomonas response regulators produced in an *E. coli* heterologous expression host exhibit host-derived post-translational phosphorylation

Megan E. Garber*[1,2], Rodrigo Fregoso*[2,3], Julie Lake[2,4], Anne Kakouridis[2], Aindrila Mukhopadhyay**[1,2]

Author Affiliations

1. University of California, Berkeley Department of Comparative Biochemistry
2. Lawrence Berkeley National Laboratory, Biological Systems and Engineering Division
3. University of California, Berkeley Department of Chemistry
4. University of California, Berkeley Department of Plant and Microbial Biology

* Equal contribution

** Correspondence: amukhopadhyay@lbl.gov

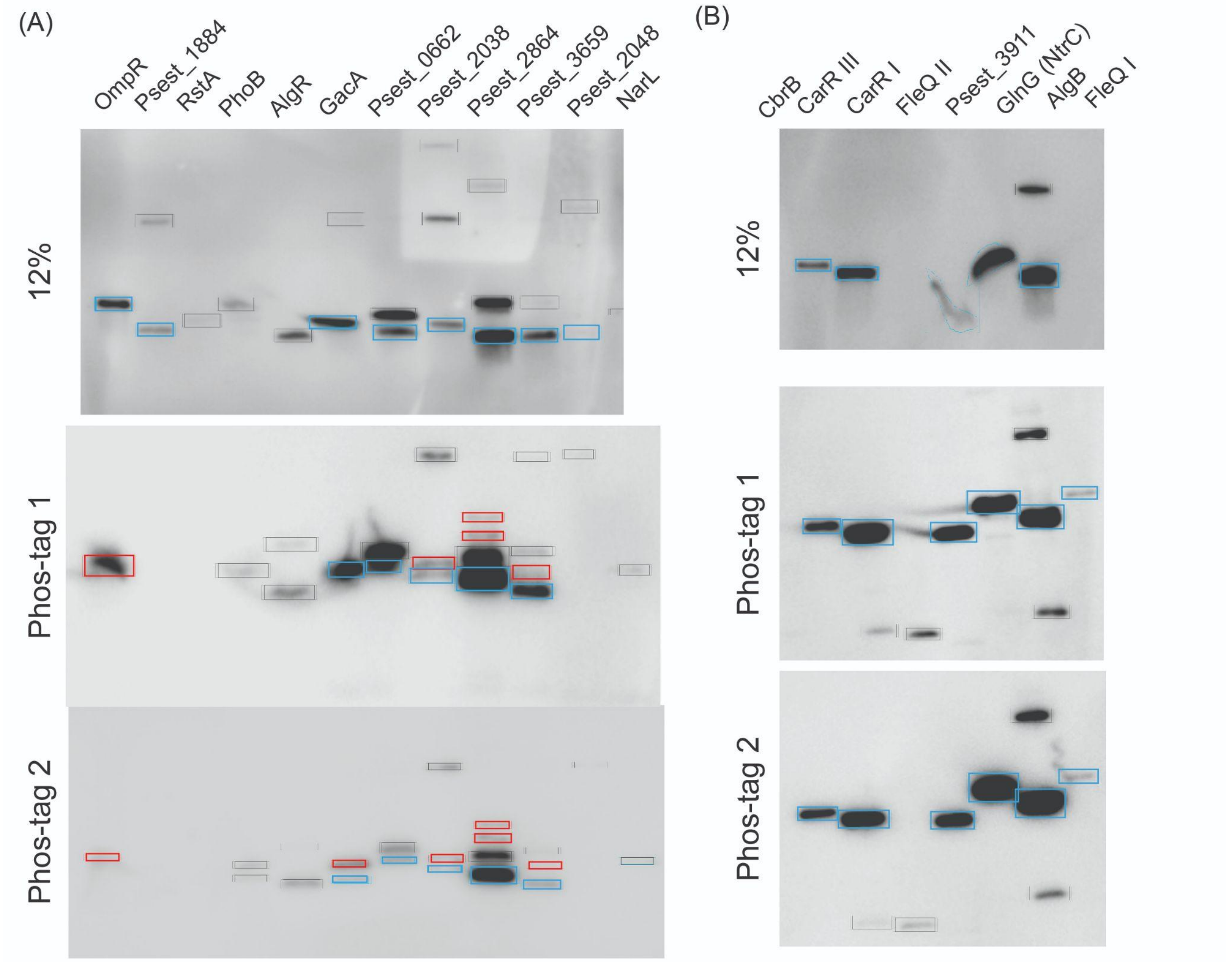

SF1 *Systematic characterization of phosphorylation state of heterologously expressed RRs* 12% and phos-tag gels of (A) OmpR-like or NarL-like RRs and (B) NtrC-like RRs from *Pseudomonas stutzeri* RCH2. Blue boxes around unphosphorylated protein, red boxes around shifted phosphorylate proteins in phos-tag gels, black boxes around background bands.

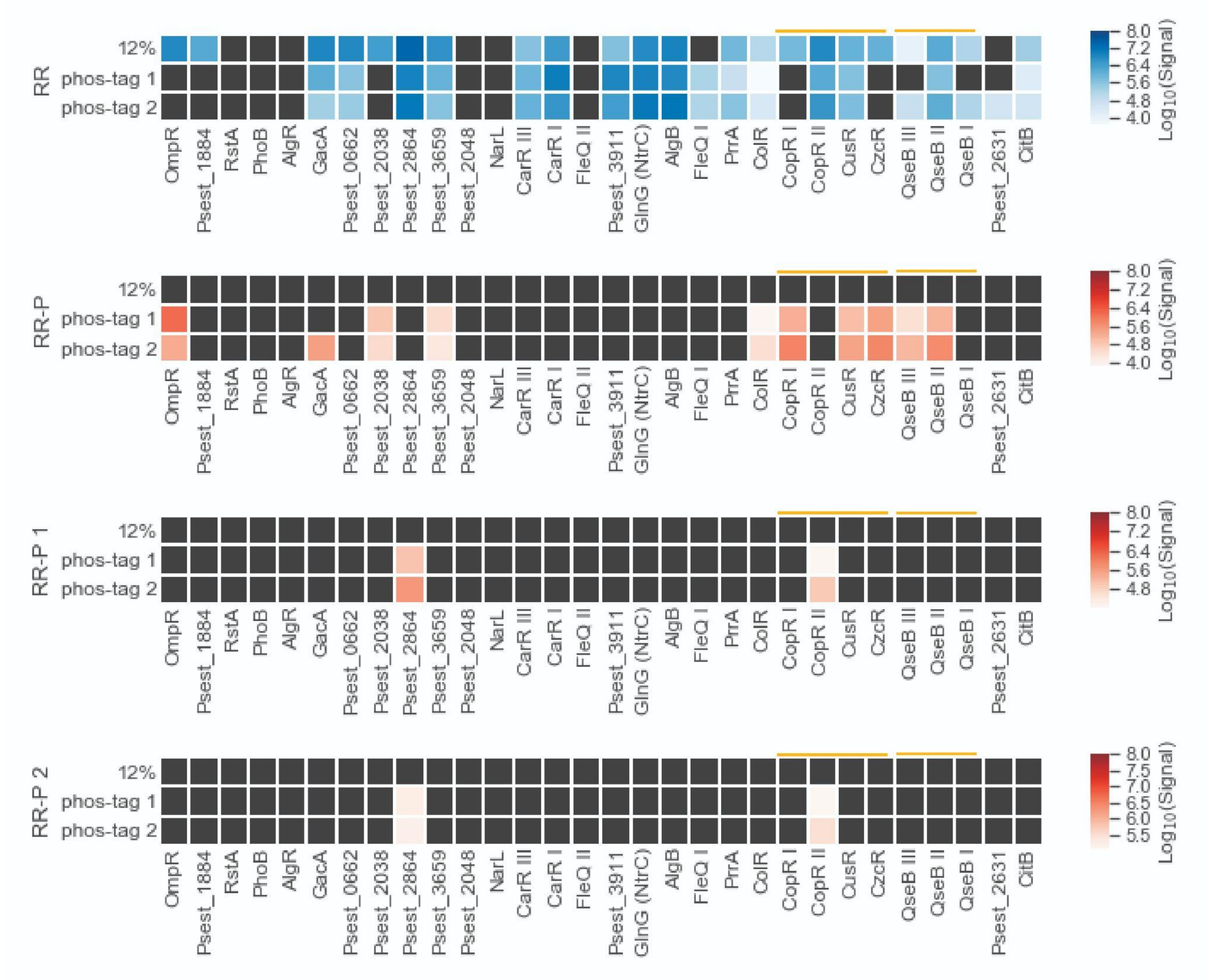

SF2: *Quantification for systematic characterization of phosphorylation state of heterologously expressed RRs* graphical quantification of bands in 12% or phos-tag gels for each RR characterized from *P. stutzeri* RCH2. Blue heatmap shows quantification for non-phosphorylated bands (RR), red heatmap shows quantification for shifted, phosphorylated bands (RR-P). Plots labeled as RR-P 1 and RR-P 2 show the quantification of two distinctive shifted bands identified as two subpopulations of phosphorylated RRs that did not appear in the 12% gel. Paralogous RRs referenced in text are indicated by yellow bars.

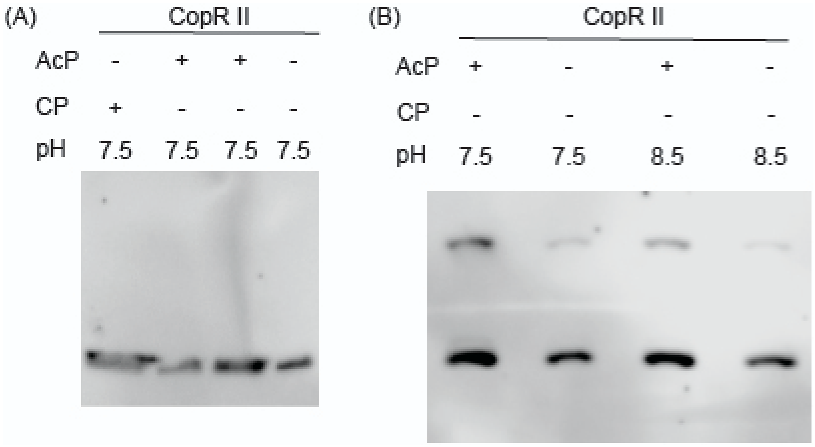

SF3: *Recombinant CopR II has a partially phosphorylated population when cultivated in auto-induction media* Recombinant CopR II was purified from expression strains cultivated in auto-induction media. Phospho-donors were added exogenously to purified protein with buffer pH 7.5 or 8.5. Proteins were resolved with (A) 12% (B) phos-tag gels. Quantitation can be referred to in ST1.

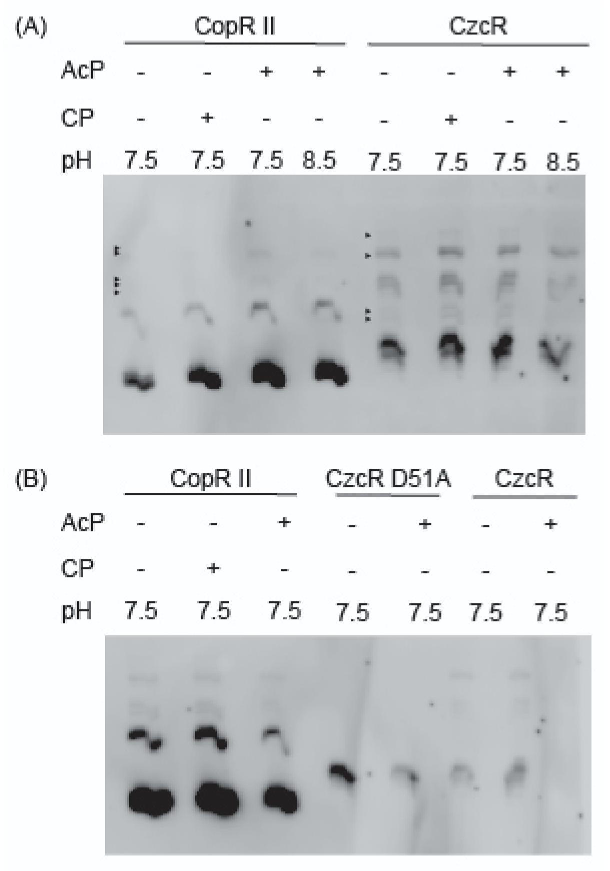

SF4 *Recombinant CopR II and CzcR has a partially phosphorylated population when cultivated in TB media* (A) Recombinant CopR II and CzcR were purified from expression strains cultivated in TB media. Phospho-donors were added exogenously to purified protein with buffer pH 7.5 or 8.5. Proteins were resolved with phos-tag gels. (B) Recombinant CopR II, CzcR D51A and CzcR were purified from expression strains cultivated in TB media. Phospho-donors were added exogenously to purified protein with buffer pH 7.5. Proteins were resolved with phos-tag gels. CzcR D51A does not have a phosphorylated population represented by a band shift. Quantitation can be referred to in Figure 2 and ST2.

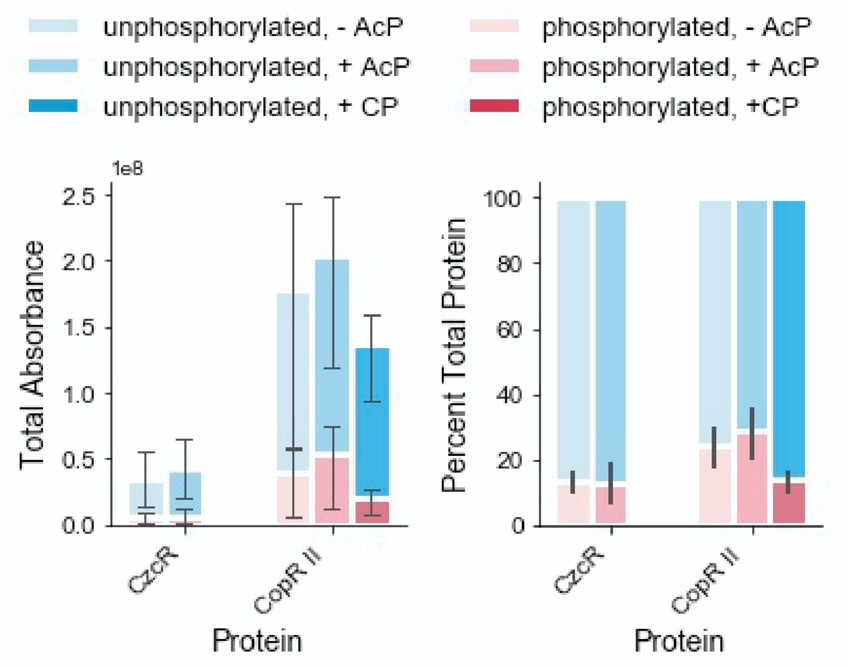

SF5 *The effect of exogenously added phospho-donors on CzcR and CopR II* Quantitation of unphosphorylated (blue) and phosphorylated (red) bands for CzcR and CopR II with and without phospho-donors AcP or CP. Bars and error bars represent the averages and standard errors between the average of two technical replicates.

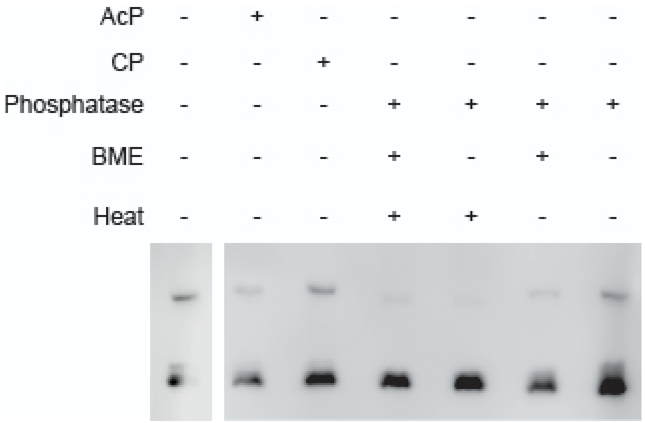

SF6 *Recombinant CopR II is dephosphorylated when under denaturing conditions* Recombinant CopR II was purified from expression strains cultivated in TB media. Phospho-donors or dephosphorylation reagents were added exogenously to purified protein with buffer pH 7.5. For heat conditions, recombinant proteins were denatured at 100 ˚C for 10 minutes. Proteins were resolved with phos-tag gels. Quantitation can be referred to in ST3.

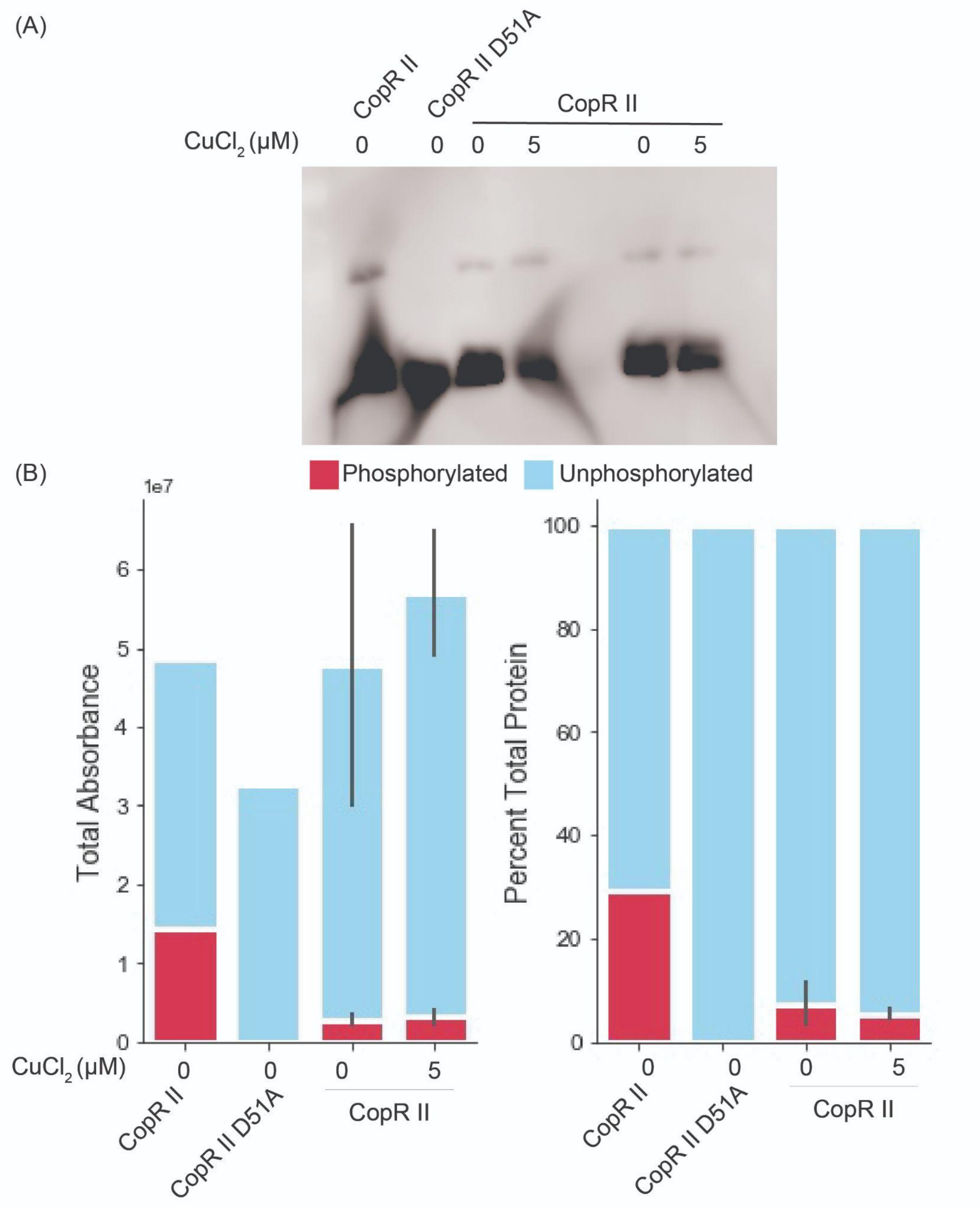

SF7 *Cultivation with copper chloride does not impact the phosphorylated population of recombinant CopR II* phos-tag western blot (A) and quantitation by total and percent absorbance (B) for CopR II cultivated in TB media with and without $CuCl_2$. The first two lanes of CopR II and CopR II D51A were cultivated in a prior batch without $CuCl_2$ and were stored at cryogenic temperatures for greater than 2 weeks before electrophoretic separation. Bars and error bars represent the averages and standard errors between the average of two technical replicates.

Supplementary Tables

ST1: *Recombinant CopR II has a partially phosphorylated population when cultivated in auto-induction media* Recombinant CopR II was purified from expression strains cultivated in auto-induction media. Phospho-donors were added exogenously to purified protein with buffer pH 7.5 or 8.5. Proteins were resolved with 12% or phos-tag gels. Absorbance and % total absorbance are reported for phosphorylated (P) and unphosphorylated (U) bands. Annotated images can be referred to in SF3.

Gel	Media	Protein	Condition	Abs (U) ÷ 10^6	Abs(P) ÷ 10^6	Abs (T) ÷ 10^6	%U	%P	Figure
12%	AI	CopR II	CP pH 7.5	2.29	0	2.29	100	0	SF3A
12%	AI	CopR II	AcP pH 7.5	1.32	0	1.32	100	0	SF3A
12%	AI	CopR II	AcP pH 8.5	3.31	0	3.31	100	0	SF3A
12%	AI	CopR II	- pH 7.5	1.59	0	1.59	100	0	SF3A
phos-tag	AI	CopR II	AcP pH 7.5	8.36	2.88	11.24	74.37	25.63	SF3B
phos-tag	AI	CopR II	- pH 7.5	4.30	0.65	4.95	86.92	13.08	SF3B
phos-tag	AI	CopR II	AcP pH 8.5	6.14	1.45	7.59	80.9	19.1	SF3B
phos-tag	AI	CopR II	- pH 8.5	3.49	0.57	4.06	86.07	13.93	SF3B

ST2: *Recombinant CopR II and CzcR has a partially phosphorylated population when cultivated in TB media* Recombinant CopR II, CzcR D51A and CzcR were purified from expression strains cultivated in TB media. Phospho-donors were added exogenously to purified protein with buffer pH 7.5 or 8.5. Proteins were resolved with phos-tag gels. Absorbance and % total absorbance are reported for phosphorylated (P) and unphosphorylated (U) bands. Annotated images can be referred to in SF4.

**Media**	**Protein**	**Condition**	**Abs (U) ÷ 10^6**	**Abs(P) ÷ 10^6**	**Abs (T) ÷ 10^6**	**%U**	**%P**	**Figure**
TB	CopR II	AcP pH 8.5	102.85	12.77	115.62	88.95	11.05	SF4
TB	CzcR D51A	- pH 7.5	63.19	0.00	63.19	100	0	SF4
TB	CzcR D51A	AcP pH 7.5	21.48	0.00	21.48	100	0	SF4

ST3: *Recombinant CopR II is dephosphorylated when under denaturing conditions* Recombinant CopR II was purified from expression strains cultivated in TB media. Phospho-donors or dephosphorylation reagents were added exogenously to purified protein with buffer pH 7.5. For heat conditions, recombinant proteins were denatured at 100 ˚C for 10 minutes. Proteins were resolved with phos-tag gels. Absorbance and % total absorbance are reported for phosphorylated (P) and unphosphorylated (U) bands. Annotated image can be referred to in SF5.

**Media**	**Protein**	**Condition**	**Abs (U) ÷ 10^6**	**Abs(P) ÷ 10^6**	**Abs (T) ÷ 10^6**	**%U**	**%P**	**Figure**
TB	CopR II	- pH 7.5	10.88	5.65	16.53	65.83	34.17	SF5
TB	CopR II	AcP pH 7.5	13.86	3.52	17.38	79.74	20.26	SF5
TB	CopR II	CP pH 7.5	43.87	10.46	54.33	80.74	19.26	SF5
TB	CopR II	Phosphatase + BME + Heat	54.96	2.21	57.17	96.13	3.87	SF5
TB	CopR II	Phosphatase + Heat	62.17	1.62	63.79	97.47	2.53	SF5
TB	CopR II	Phosphatase + BME	41.41	3.26	44.67	92.71	7.29	SF5
TB	CopR II	Phosphatase	84.96	12.29	97.25	87.37	12.63	SF5

ST4: *Recombinant protein expression strains used in this study*

Protein Name	Locus ID	Expected Band Size	TCS family	JBEI Public registry Part ID
OmpR	Psest_3995	27	OmpR	JPUB_019383
Psest_1884	Psest_1884	24	OmpR	JPUB_019382
RstA	Psest_2730	27	OmpR	JPUB_019374
PhoB	Psest_3845	25	OmpR	JPUB_019381
AlgR	Psest_3749	28	LytR	JPUB_019371
GacA	Psest_2260	23	NarL	JPUB_019393
Psest_0662	Psest_0662	23	NarL	JPUB_019378
Psest_2038	Psest_2038	24	NarL	JPUB_019397
Psest_2864	Psest_2864	23	NarL	JPUB_019375
Psest_3659	Psest_3659	22	NarL	JPUB_019389
Psest_2048	Psest_2048	24	NarL	JPUB_019391
NarL	Psest_3491	22	NarL	JPUB_019369
CbrB	Psest_0999	51	NtrC	JPUB_019372
CarR III	Psest_4267	50	NtrC	JPUB_019395
CarR I	Psest_4132	49	NtrC	JPUB_019390
FleQ II	Psest_1767	50	FleQ	JPUB_019392
Psest_3911	Psest_3911	51	NtrC	JPUB_019396
GlnG (NtrC)	Psest_3932	53	NtrC	JPUB_019388
AlgB	Psest_0192	49	NtrC	JPUB_019386
FleQ I	Psest_1765	55	FleQ	JPUB_019387
PrrA	Psest_3338	20	NtrX	JPUB_019370
PhoP	Psest_3883	25	OmpR	JPUB_019394
ColR	Psest_3465	25	OmpR	JPUB_019385
CopR I	Psest_0581	25	OmpR	JPUB_019366
CopR II	Psest_1598	25	OmpR	JPUB_019367
CusR	Psest_1185	25	OmpR	JPUB_019377

CzcR	Psest_0611	25	OmpR	JPUB_019368
QseB III	Psest_3533	24	OmpR	JPUB_019373
QseB II	Psest_3476	24	OmpR	JPUB_019379
QseB I	Psest_1602	24	OmpR	JPUB_019380
Psest_2631	Psest_2631	33	AraC	JPUB_019376
CitB	Psest_3922	27	N.A.	JPUB_019384
CopR II D51A	Psest_1598	25	OmpR	JPUB_019399
CzcR D51A	Psest_0611	25	OmpR	JPUB_019398

ST5: *Primers for construction of E. coli BL21 (DE3) ΔcusS*

**Primer Name**	**Sequence**
F_CusS_recomb	CTTTGAGCCGAAGCTAATTCAGACCGTGCGCGGCGTGGGTTACATGCTTGattccggggatccgtcgacc
R_CusS_recomb	GGTTATAAAAGTTGCCGTTTGCTGAAGGATTAAGCGGGTAATGTGATAACtgtaggctggagctgcttcg
F_CusS_ColPCR	AGCGACACTAACGCCATTGA
R_CusS_ColPCR	CCGCTCCACCCTGATAACAT

ST6: *Raw file metadata*

File	Figure Number	Data loc	Image desc.
Image01-1.tif	F1	F1	phos-tag
Image01-2.tif	F1	F1	phos-tag
Image01-3.tif	F1	F1	12%
Image02-1.tif	SF2	F1	phos-tag
Image02-2.tif	SF2	F1	12%
Image03-1.tif	SF1	F1	phos-tag
Image03-2.tif	SF1	F1	phos-tag
Image03-3.tif	SF1	F1	12%
Image04.tif	F2	F2	phos-tag
Image05.tif	F3	F3	phos-tag
Image06-1.tif	F4	F4	phos-tag
Image06-2.tif	F4		12%
Image07.tif	SF3	ST1	phos-tag
Image08.tif	SF4	F2	phos-tag
Image09.tif	SF4	F2	phos-tag
Image10.tif	SF5	ST3	phos-tag
Image11.tif	SF6	SF6	phos-tag
Image12.tif	SF3	ST1	12%
