# Supplementary material for: Pseudomonas response regulators produced in an E. coli heterologous expression host exhibit host-derived post-translational phosphorylation
Source: Sci Rep. 2022 Jun 20;12:10336. doi: 10.1038/s41598-022-13525-2 (PMC9209504; doi:10.1038/s41598-022-13525-2)
Supplement: Supplementary file 2 — Supplementary Information 2. [file 41598_2022_13525_MOESM2_ESM.docx]

**Figure_1_B_12%**

| Column Number | Sample Name |
| --- | --- |
| 1 | PrrA |
| 2 | PhoP |
| 3 | ColR |
| 4 | CopR I |
| 5 | CopR II |
| 6 | CusR |
| 7 | CzcR |
| 8 | QseB III |
| 9 | QseB II |
| 10 | QseB I |
| 11 | Psest_2631 |
| 12 | CitB |

**Figure_1_b_Phos-tag_1**

| Column Number | Sample Name |
| --- | --- |
| 1 | PrrA |
| 2 | PhoP |
| 3 | ColR |
| 4 | CopR I |
| 5 | CopR II |
| 6 | CusR |
| 7 | CzcR |
| 8 | QseB III |
| 9 | QseB II |
| 10 | QseB I |
| 11 | Psest_2631 |
| 12 | CitB |

**Figure_1_b_Phos-tag_2**

| Column Number | Sample Name |
| --- | --- |
| 1 | PrrA |
| 2 | PhoP |
| 3 | ColR |
| 4 | CopR I |
| 5 | CopR II |
| 6 | CusR |
| 7 | CzcR |
| 8 | QseB III |
| 9 | QseB II |
| 10 | QseB I |
| 11 | Psest_2631 |
| 12 | CitB |

**Figure_2_a**

| Column Number | Sample Name |
| --- | --- |
| 1 | CopR II -AcP |
| 2 | CopR II +AcP |
| 3 | CopR II D51A -AcP |
| 4 | CopR II D51A +AcP |
| 5 | CopR II -AcP |
| 6 | CopR II +AcP |
| 7 | CopR II D51A -AcP |
| 8 | CopR II D51A +AcP |

**Figure_3_a**

| Column Number | Sample Name |
| --- | --- |
| 1 | CopR II |
| 2 | CopR II D51A |
| 3 | CopR II 0 mM MgCl2 |
| 4 | CopR II 0.5 mM MgCl2 |
| 5 | CopR II 1 mM MgCl2 |
| 6 | CopR II 2 mM MgCl2 |
| 7 | - |
| 8 | CopR II 0 mM MgCl2 |
| 9 | CopR II 0.5 mM MgCl2 |
| 10 | CopR II 1 mM MgCl2 |
| 11 | CopR II 2 mM MgCl2 |

**Figure_4_a_Image_1**

| Column Number | Sample Name |
| --- | --- |
| 1 | Ladder |
| 2 | CopR II |
| 3 | CopR II D51A |
| 4 | WT |
| 5 | ∆pta |
| 6 | ∆ackA |
| 7 | ∆cusS |
| 8 | - |
| 9 | WT |
| 10 | ∆pta |
| 11 | ∆ackA |
| 12 | ∆cusS |

**Figure_4_a_Image_2**

| Column Number | Sample Name |
| --- | --- |
| 1 | Ladder |
| 2 | CopR II |
| 3 | CopR II D51A |
| 4 | - |
| 5 | WT |
| 6 | ∆pta |
| 7 | ∆ackA |
| 8 | ∆cusS |
| 9 | - |
| 10 | CopR II |
| 11 | CopR II D51A |
| 12 | WT |
| 13 | ∆pta |
| 14 | ∆ackA |
| 15 | ∆cusS |

**SF_1_a_12%**

| Column Number | Sample Name |
| --- | --- |
| 1 | OmpR |
| 2 | Psest_1884 |
| 3 | RstA |
| 4 | PhoB |
| 5 | AlgR |
| 6 | GacA |
| 7 | Psest_0662 |
| 8 | Psest_2038 |
| 9 | Psest_2864 |
| 10 | Psest_3659 |
| 11 | Psest_2048 |
| 12 | NarL |

**SF_1_a_Phos-tag_1**

| Column Number | Sample Name |
| --- | --- |
| 1 | OmpR |
| 2 | Psest_1884 |
| 3 | RstA |
| 4 | PhoB |
| 5 | AlgR |
| 6 | GacA |
| 7 | Psest_0662 |
| 8 | Psest_2038 |
| 9 | Psest_2864 |
| 10 | Psest_3659 |
| 11 | Psest_2048 |
| 12 | NarL |

**SF_1_a_Phos-tag_2**

| Column Number | Sample Name |
| --- | --- |
| 1 | OmpR |
| 2 | Psest_1884 |
| 3 | RstA |
| 4 | PhoB |
| 5 | AlgR |
| 6 | GacA |
| 7 | Psest_0662 |
| 8 | Psest_2038 |
| 9 | Psest_2864 |
| 10 | Psest_3659 |
| 11 | Psest_2048 |
| 12 | NarL |

**SF_1_b_12%**

| Column Number | Sample Name |
| --- | --- |
| 1 | CbrB |
| 2 | CarR III |
| 3 | CarR I |
| 4 | FleQ II |
| 5 | Psest_3911 |
| 6 | GlnG (NtrC) |
| 7 | AlgB |
| 8 | FleQ I |

**SF_1_b_Phos-tag_1_and_Phos-tag_2**

| Column Number | Sample Name |
| --- | --- |
| 1 | CbrB |
| 2 | CarR III |
| 3 | CarR I |
| 4 | FleQ II |
| 5 | Psest_3911 |
| 6 | GlnG (NtrC) |
| 7 | AlgB |
| 8 | FleQ I |
| 9 | CbrB (replicate) |
| 10 | CarR III (replicate) |
| 11 | CarR I (replicate) |
| 12 | FleQ II (replicate) |
| 13 | Psest_3911 (replicate) |
| 14 | GlnG (NtrC) (replicate) |
| 15 | AlgB (replicate) |
| 16 | FleQ I (replicate) |

**SF_3_a**

| Column Number | Sample Name |
| --- | --- |
| 1 | Ladder |
| 2 | CopR2 + CP pH = 7.5 |
| 3 | CopR2 + AP pH = 7.5 |
| 4 | CopR2 + CP +AP pH = 7.5 |
| 5 | CopR II pH = 7.5 |

**SF_3_b**

| Column Number | Sample Name |
| --- | --- |
| 1 | Ladder |
| 2 | CopR2 + AP pH = 7.5 (leakage) |
| 3 | CopR2 + AP pH = 7.5 |
| 4 | CopR2 pH = 7.5 |
| 5 | CopR2 + AP pH = 8.5 |
| 6 | CopR2 pH = 8.5 |

**SF_4_a**

| Column Number | Sample Name |
| --- | --- |
| 1 | Ladder |
| 2 | CopR2 pH = 7.5 |
| 3 | CopR2 + CP pH = 7.5 |
| 4 | CopR2 + AP pH = 7.5 |
| 5 | CopR2 + AP pH = 8.5 |
| 6 | CzcR pH = 7.5 |
| 7 | CzcR + CP pH = 7.5 |
| 8 | CzcR + AP pH = 7.5 |
| 9 | CzcR + AP pH = 8.5 |

**SF_4b**

| Column Number | Sample Name |
| --- | --- |
| 1 | Ladder |
| 2 | CopR2 pH = 7.5 |
| 3 | CopR2 + CP pH = 7.5 |
| 4 | CopR2 + AP pH = 7.5 |
| 5 | CzcR D51A pH = 7.5 |
| 6 | CzcR D51A + AP pH = 7.5 |
| 7 | CzcR pH = 7.5 |
| 8 | CzcR + AP pH = 7.5 |

**SF_6**

| Column Number | Sample Name |
| --- | --- |
| 1 | Ladder |
| 2 | CopR2 |
| 3 | - |
| 4 | CopR2 + AP |
| 5 | CopR2 + CP |
| 6 | CopR2 + Phosphatase +BME +Heat |
| 7 | CopR2 + Phosphatase +Heat |
| 8 | CopR2 +Phosphatase +BME |
| 9 | CopR2 + Phosphatase |

**SF_7_a**

| Column Number | Sample Name |
| --- | --- |
| 1 | CopR II |
| 2 | CopR II D51A |
| 3 | CopR II 0 uM CuCl2 |
| 4 | CopR II 5 uM CuCl2 |
| 5 | - |
| 6 | CopR II 0 uM CuCl2 |
| 7 | CopR II 5 uM CuCl2 |
| 8 | CopR II 0 uM CuCl2 (replicate 2) |
| 9 | CopR II 5 uM CuCl2 (replicate 2) |
| 10 | CopR II 0 uM CuCl2 (replicate 3) |
| 11 | CopR II 5 uM CuCl2 (replicate 3) |
