# Supplementary figures and images for: Pseudomonas response regulators produced in an E. coli heterologous expression host exhibit host-derived post-translational phosphorylation
Source: Sci Rep. 2022 Jun 20;12:10336. doi: 10.1038/s41598-022-13525-2 (PMC9209504; doi:10.1038/s41598-022-13525-2)

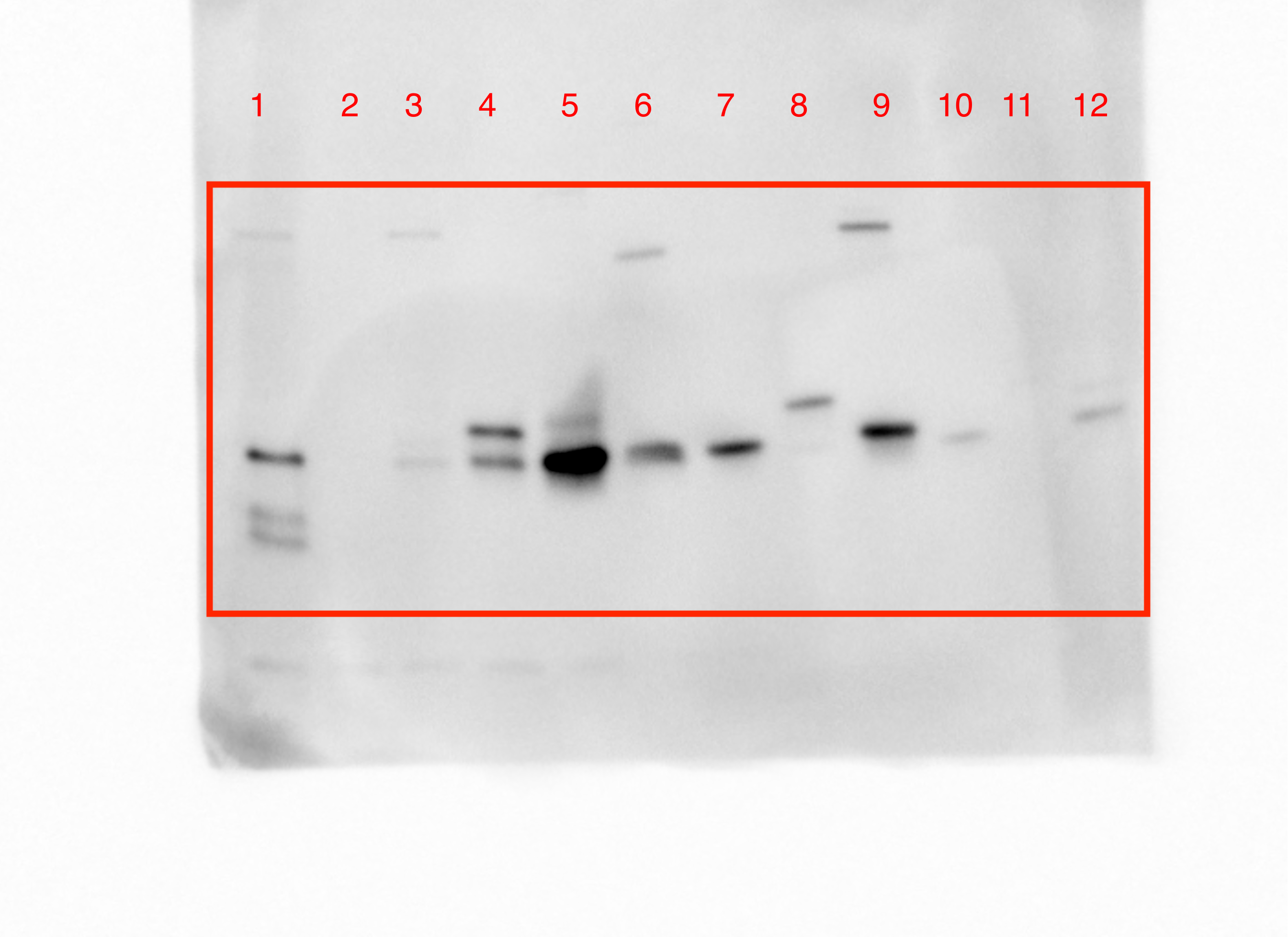

Supplement: Supplementary file 3 — Supplementary Information 3. [file 41598_2022_13525_MOESM3_ESM.tif]

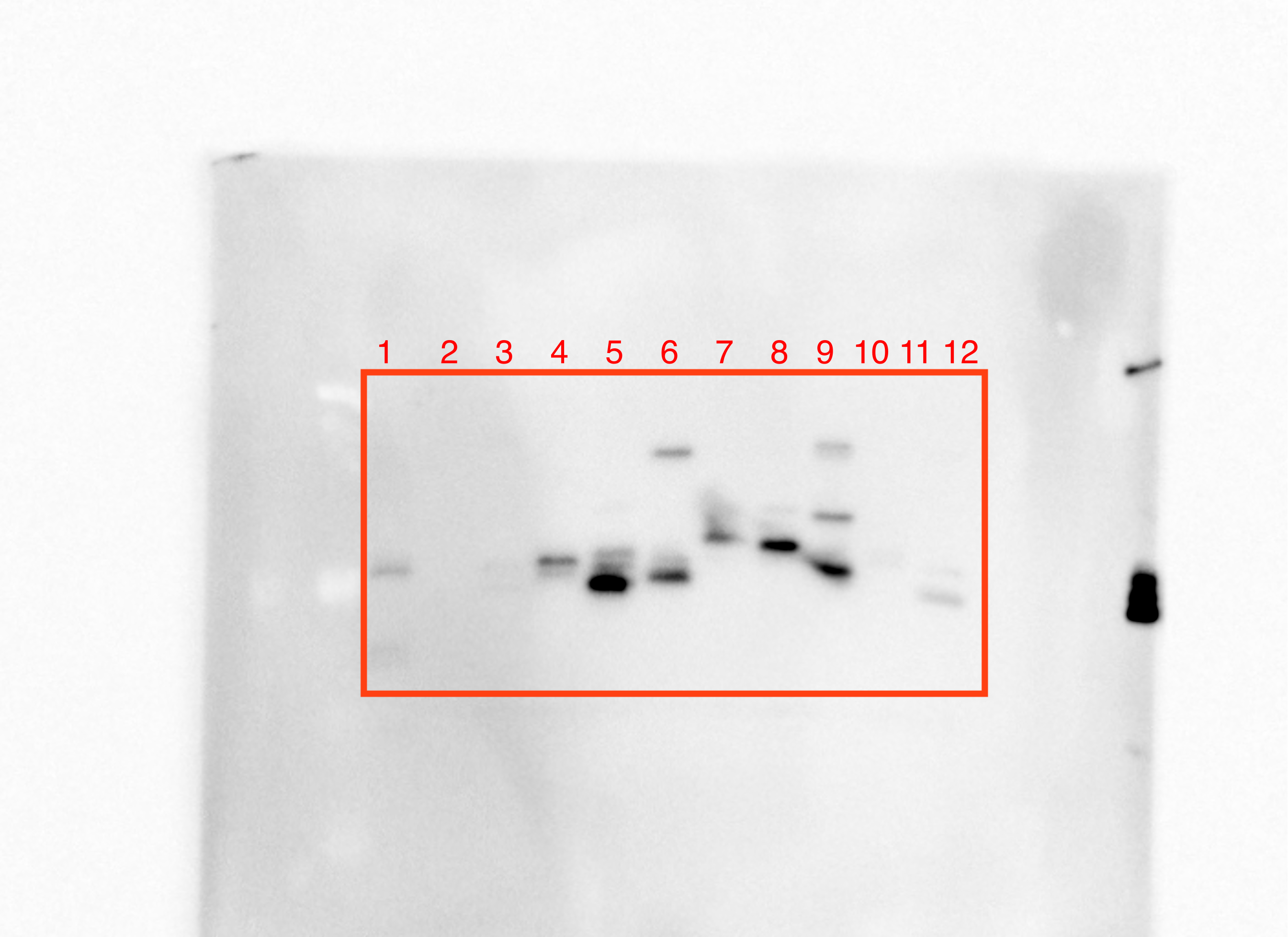

Supplement: Supplementary file 4 — Supplementary Information 4. [file 41598_2022_13525_MOESM4_ESM.tif]

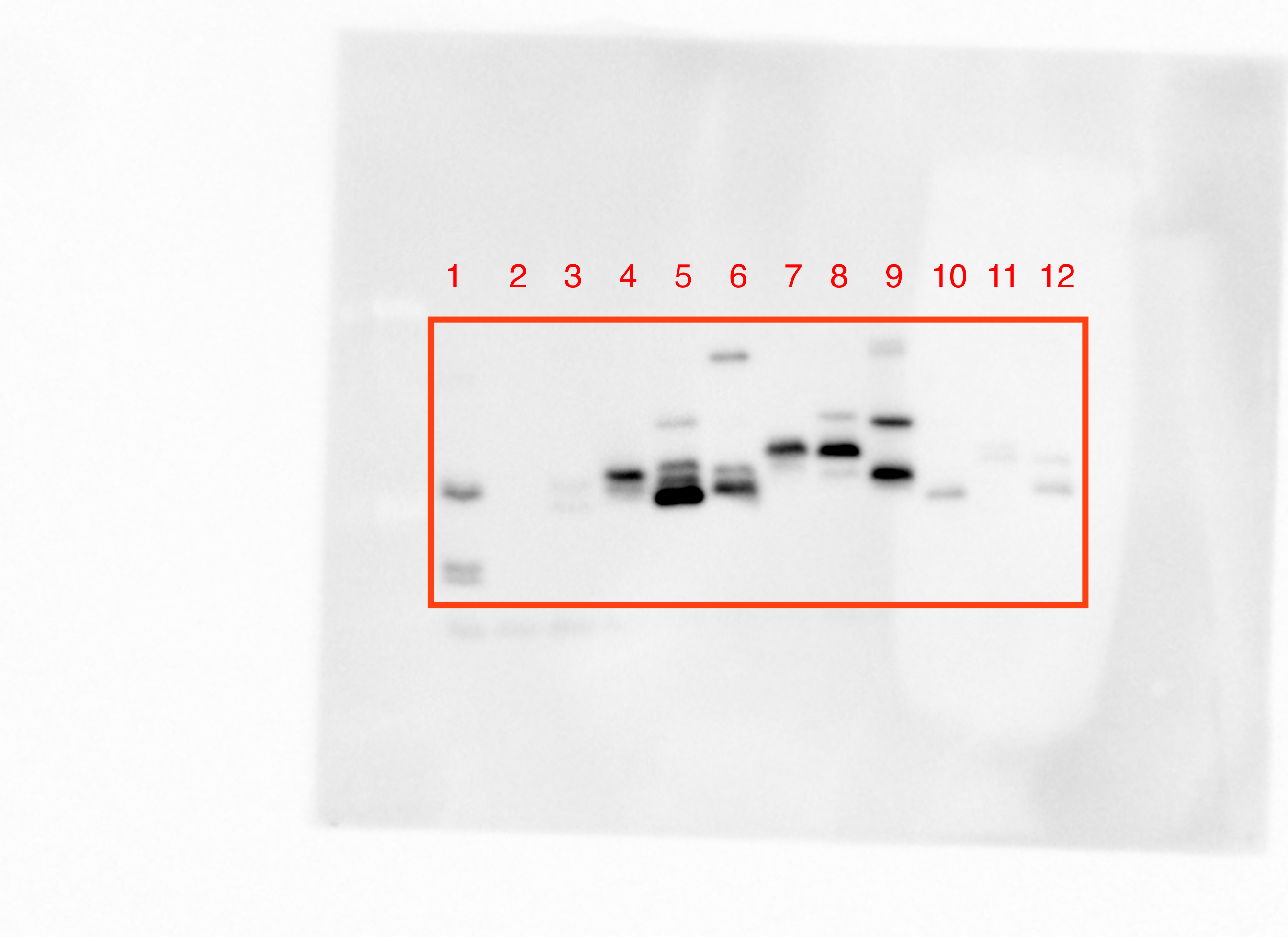

Supplement: Supplementary file 5 — Supplementary Information 5. [file 41598_2022_13525_MOESM5_ESM.tif]

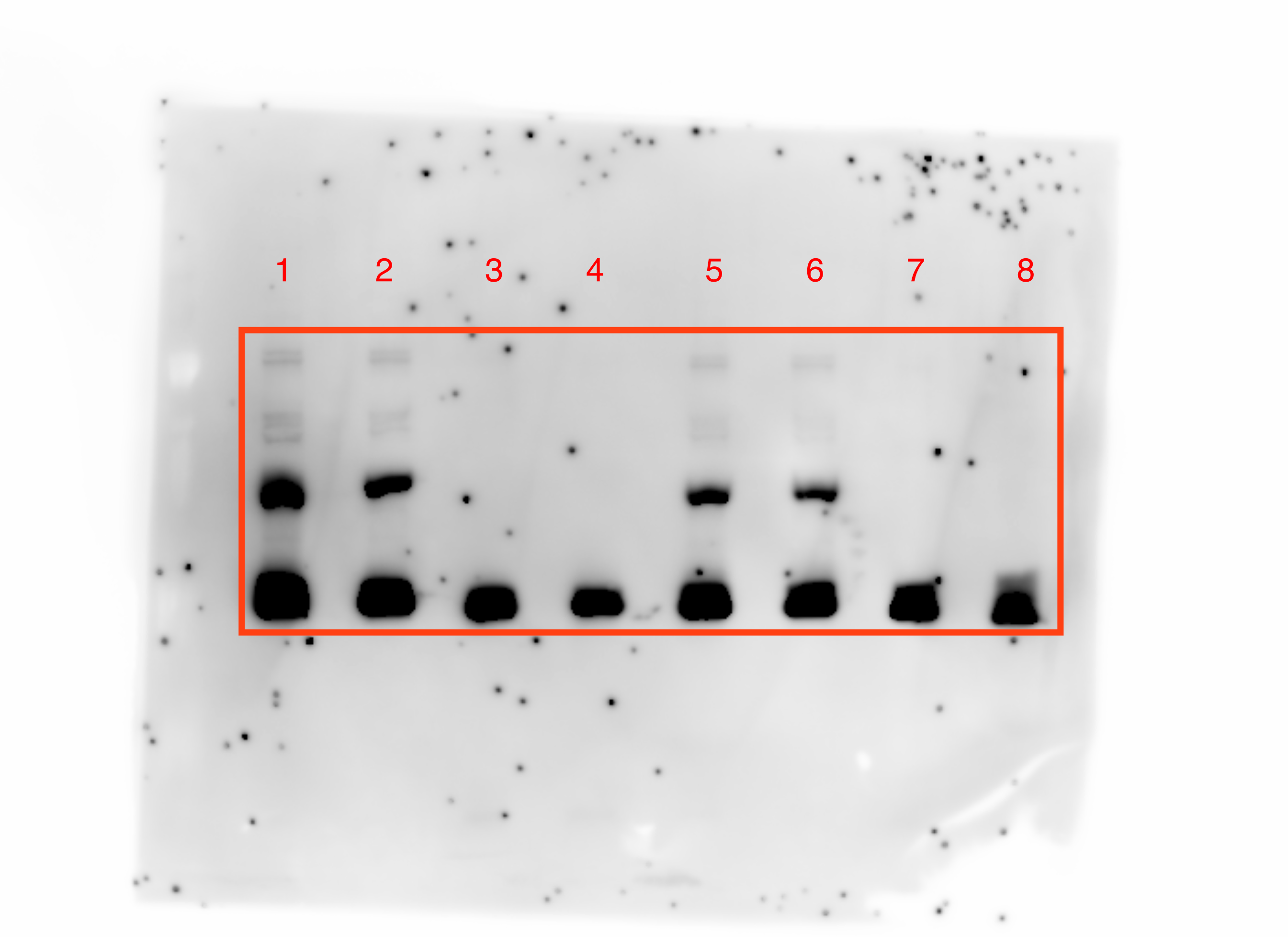

Supplement: Supplementary file 6 — Supplementary Information 6. [file 41598_2022_13525_MOESM6_ESM.tif]

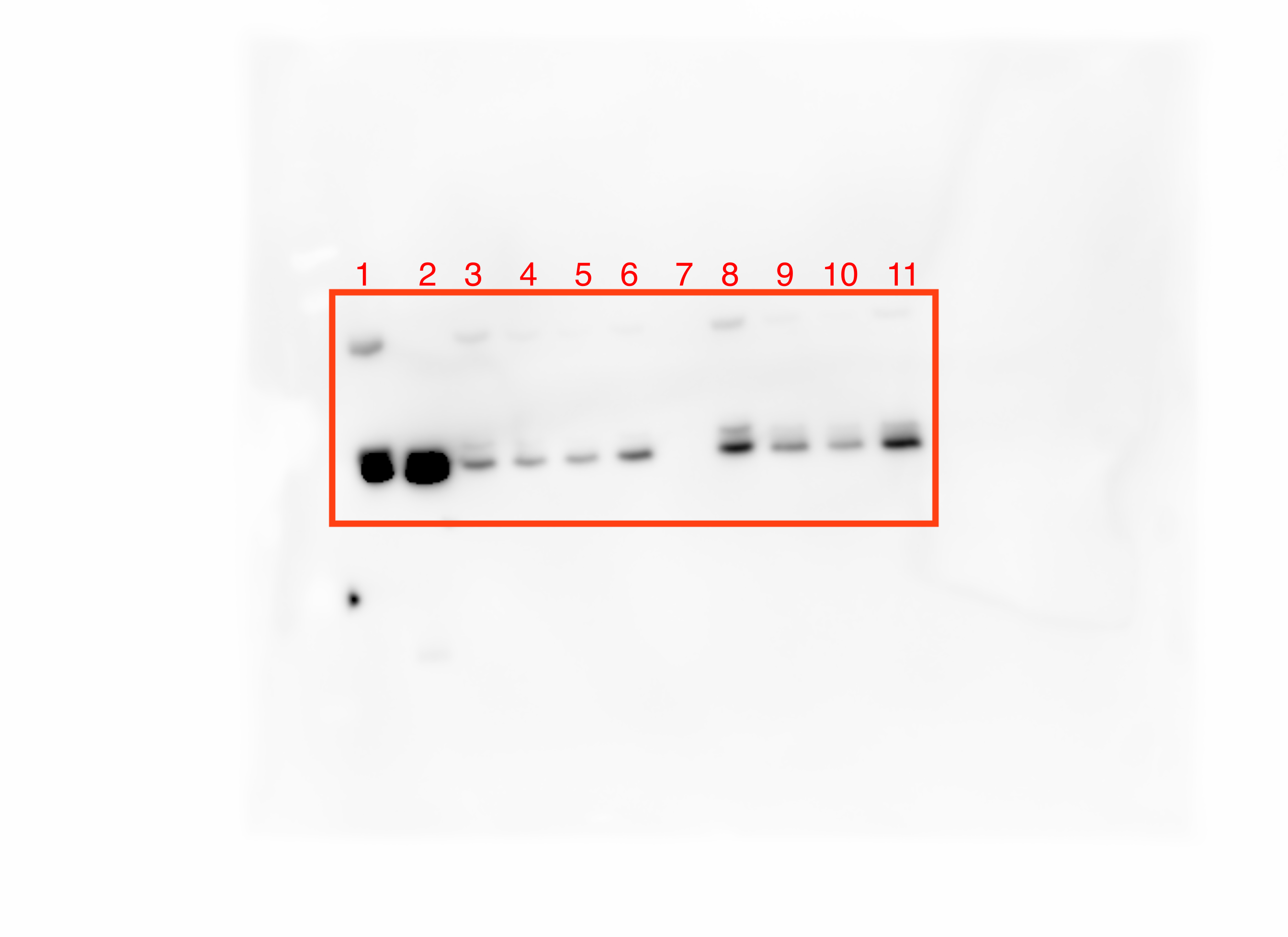

Supplement: Supplementary file 7 — Supplementary Information 7. [file 41598_2022_13525_MOESM7_ESM.tif]

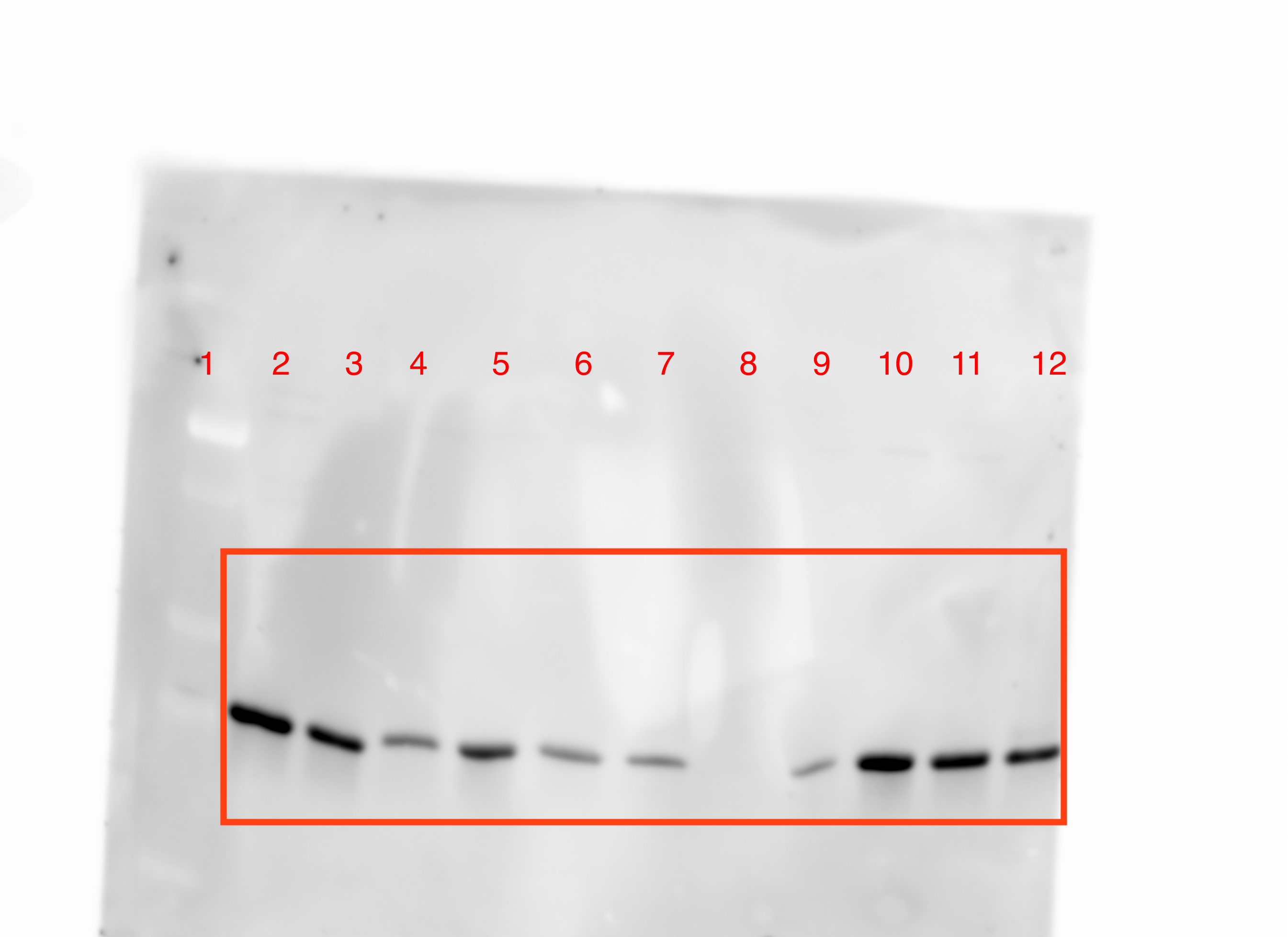

Supplement: Supplementary file 8 — Supplementary Information 8. [file 41598_2022_13525_MOESM8_ESM.tif]

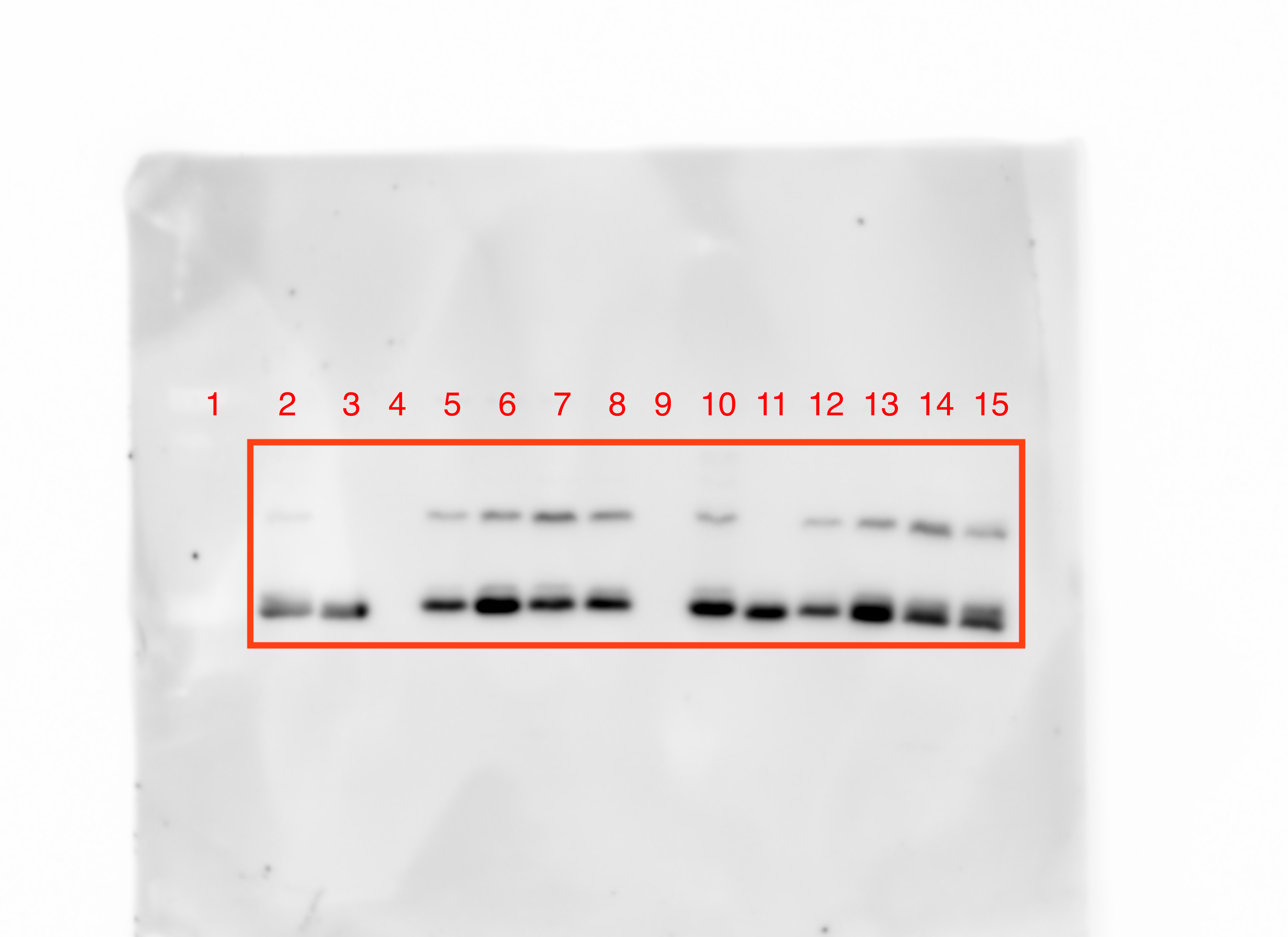

Supplement: Supplementary file 9 — Supplementary Information 9. [file 41598_2022_13525_MOESM9_ESM.tif]

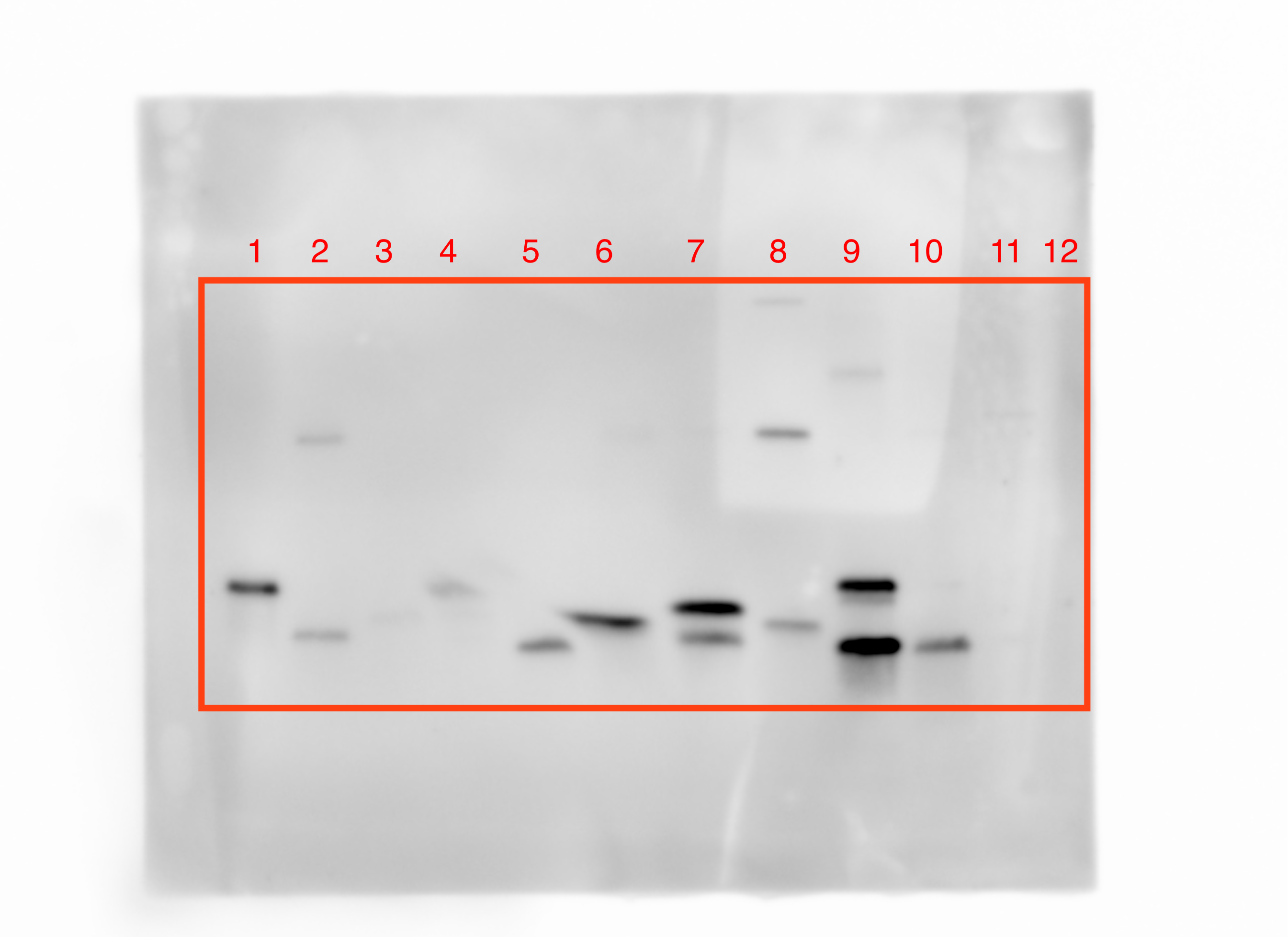

Supplement: Supplementary file 10 — Supplementary Information 10. [file 41598_2022_13525_MOESM10_ESM.tif]

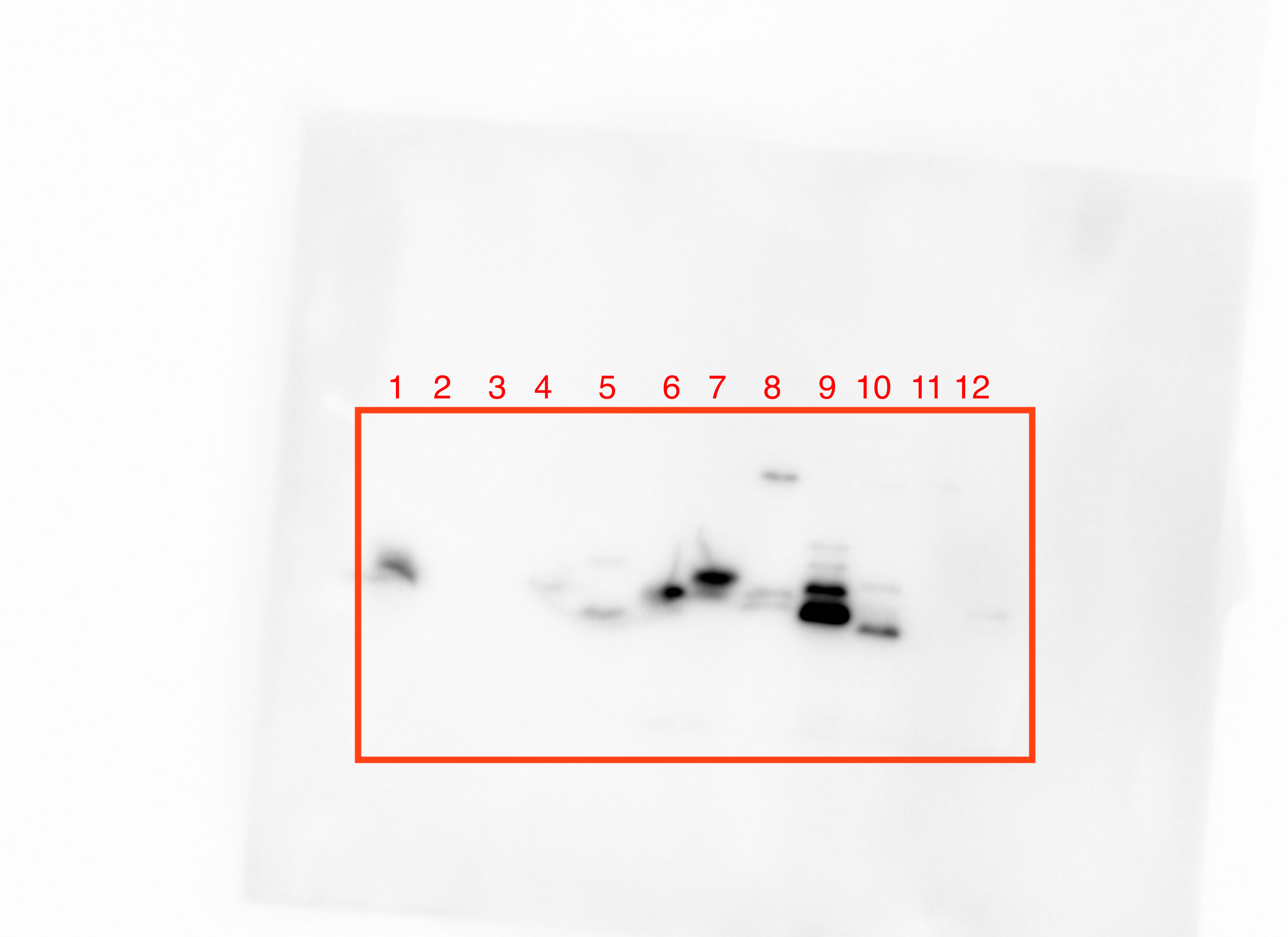

Supplement: Supplementary file 11 — Supplementary Information 11. [file 41598_2022_13525_MOESM11_ESM.tif]

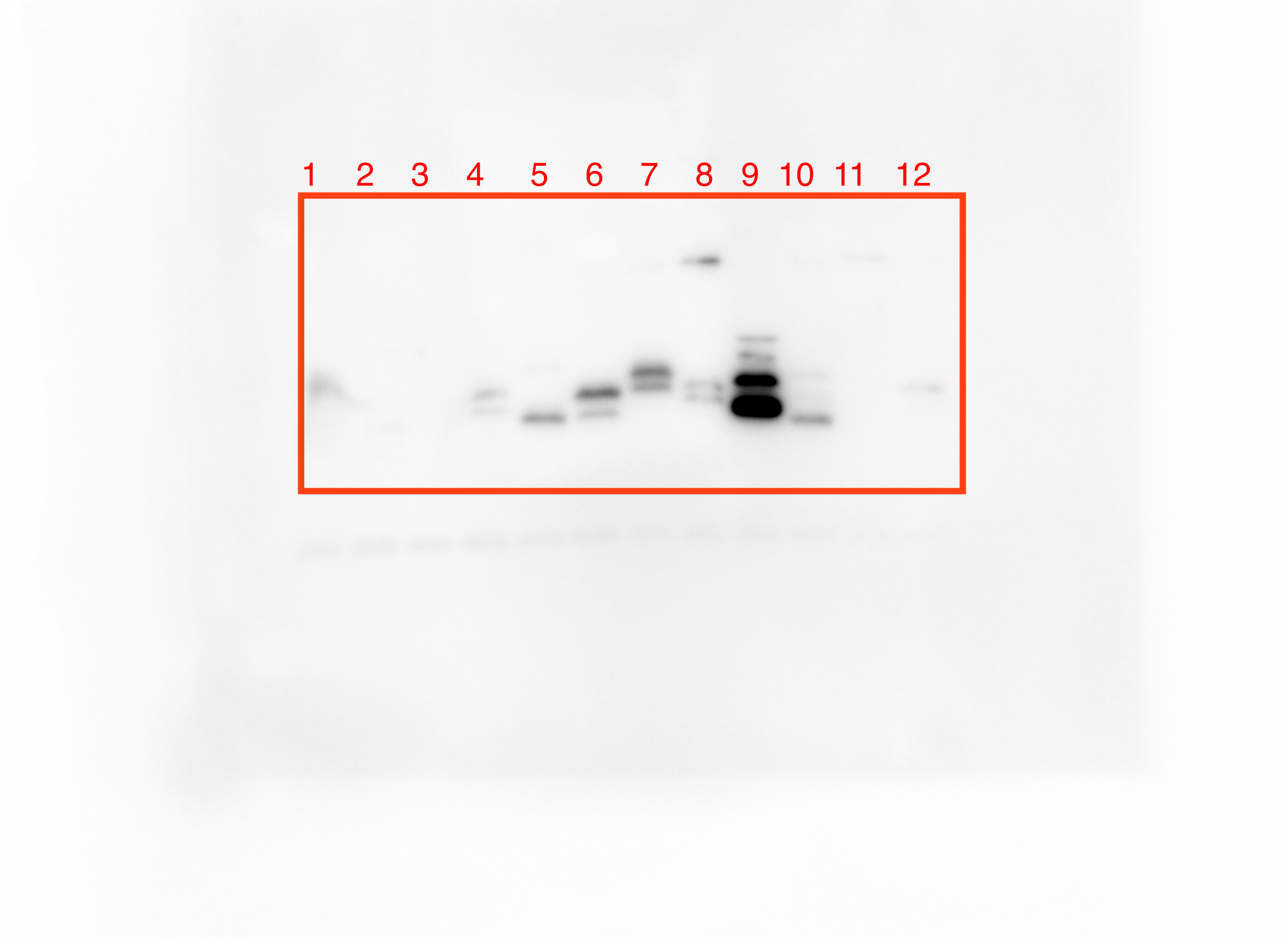

Supplement: Supplementary file 12 — Supplementary Information 12. [file 41598_2022_13525_MOESM12_ESM.tif]

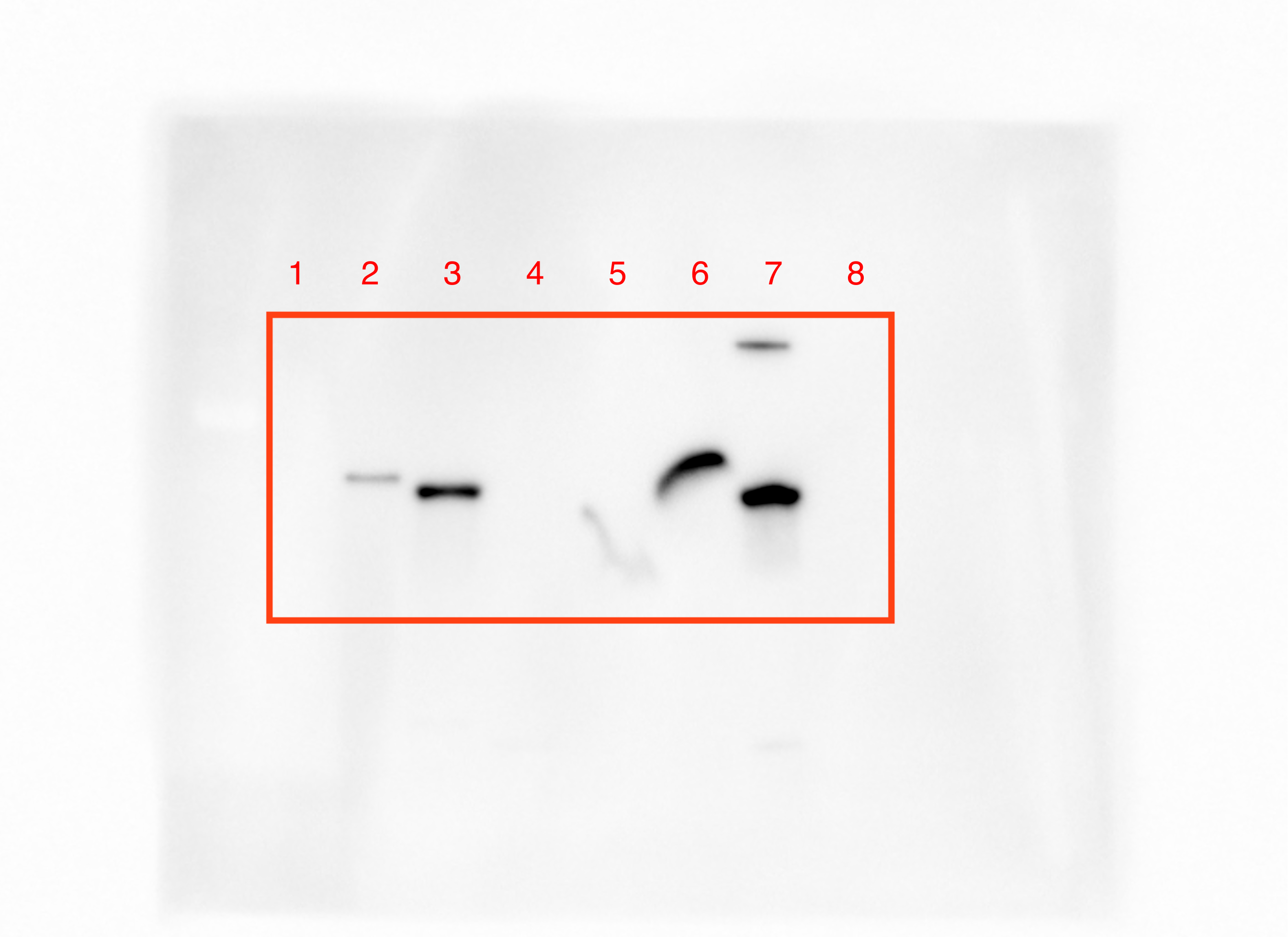

Supplement: Supplementary file 13 — Supplementary Information 13. [file 41598_2022_13525_MOESM13_ESM.tif]

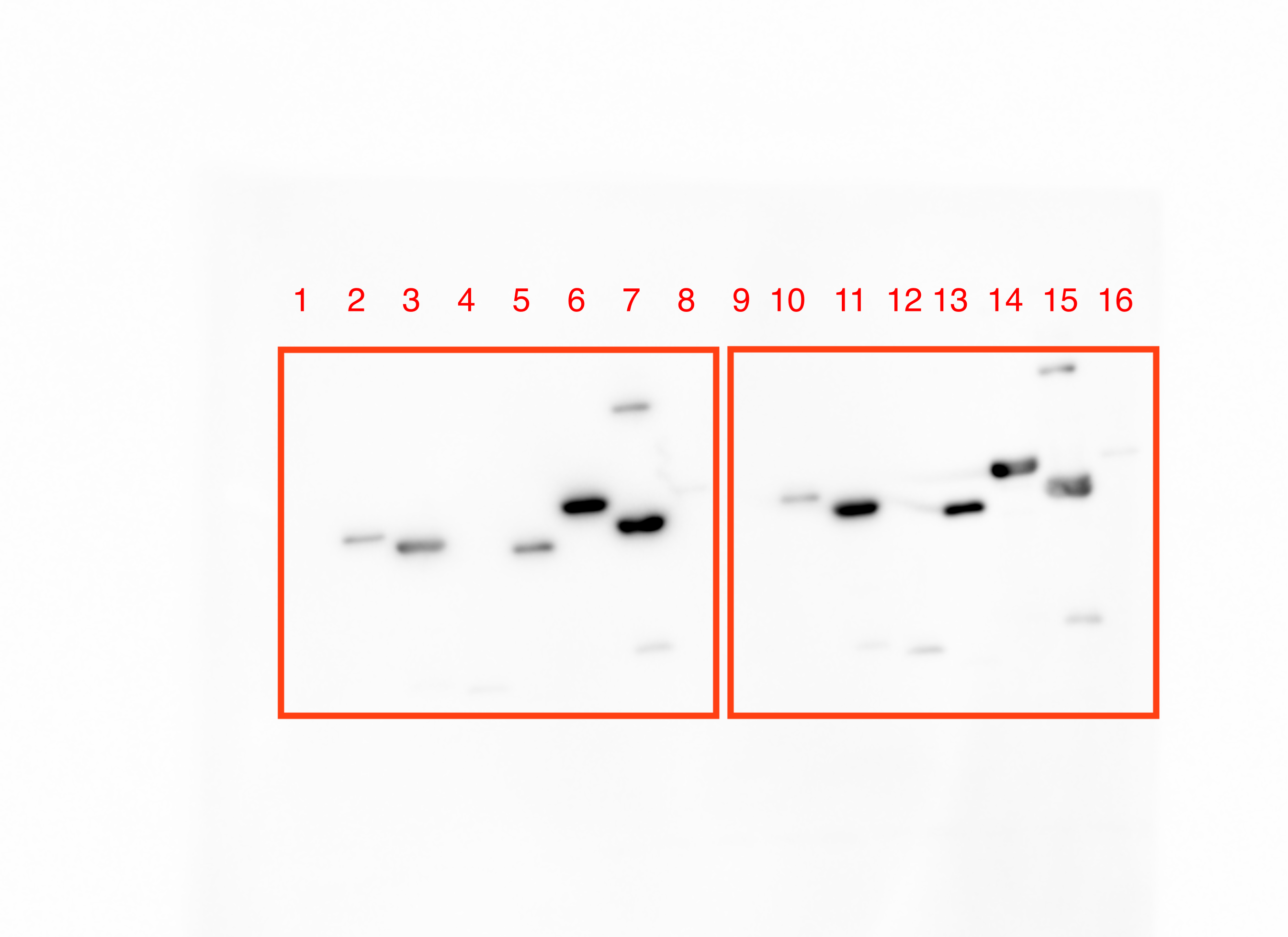

Supplement: Supplementary file 14 — Supplementary Information 14. [file 41598_2022_13525_MOESM14_ESM.tif]

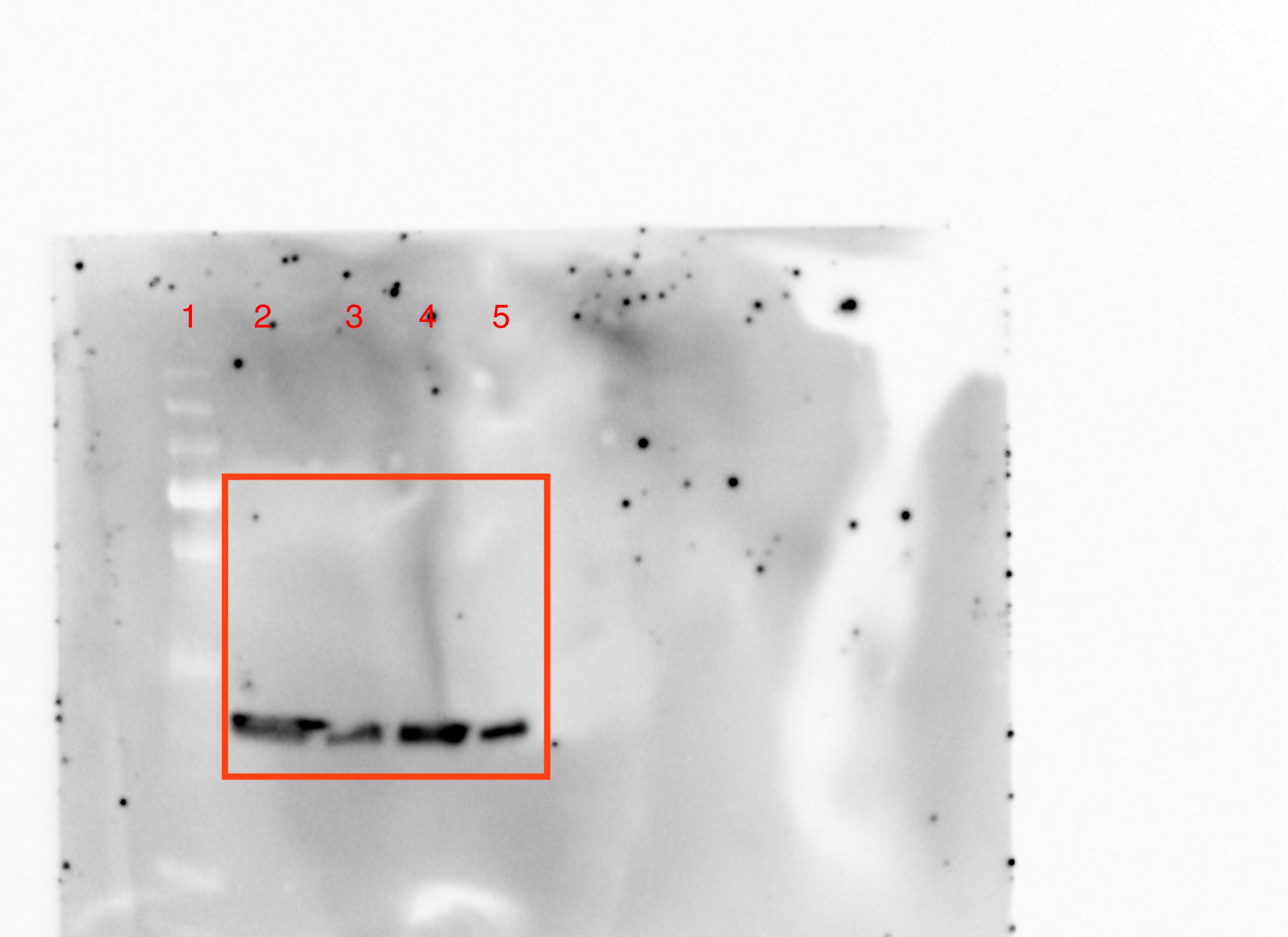

Supplement: Supplementary file 15 — Supplementary Information 15. [file 41598_2022_13525_MOESM15_ESM.tif]

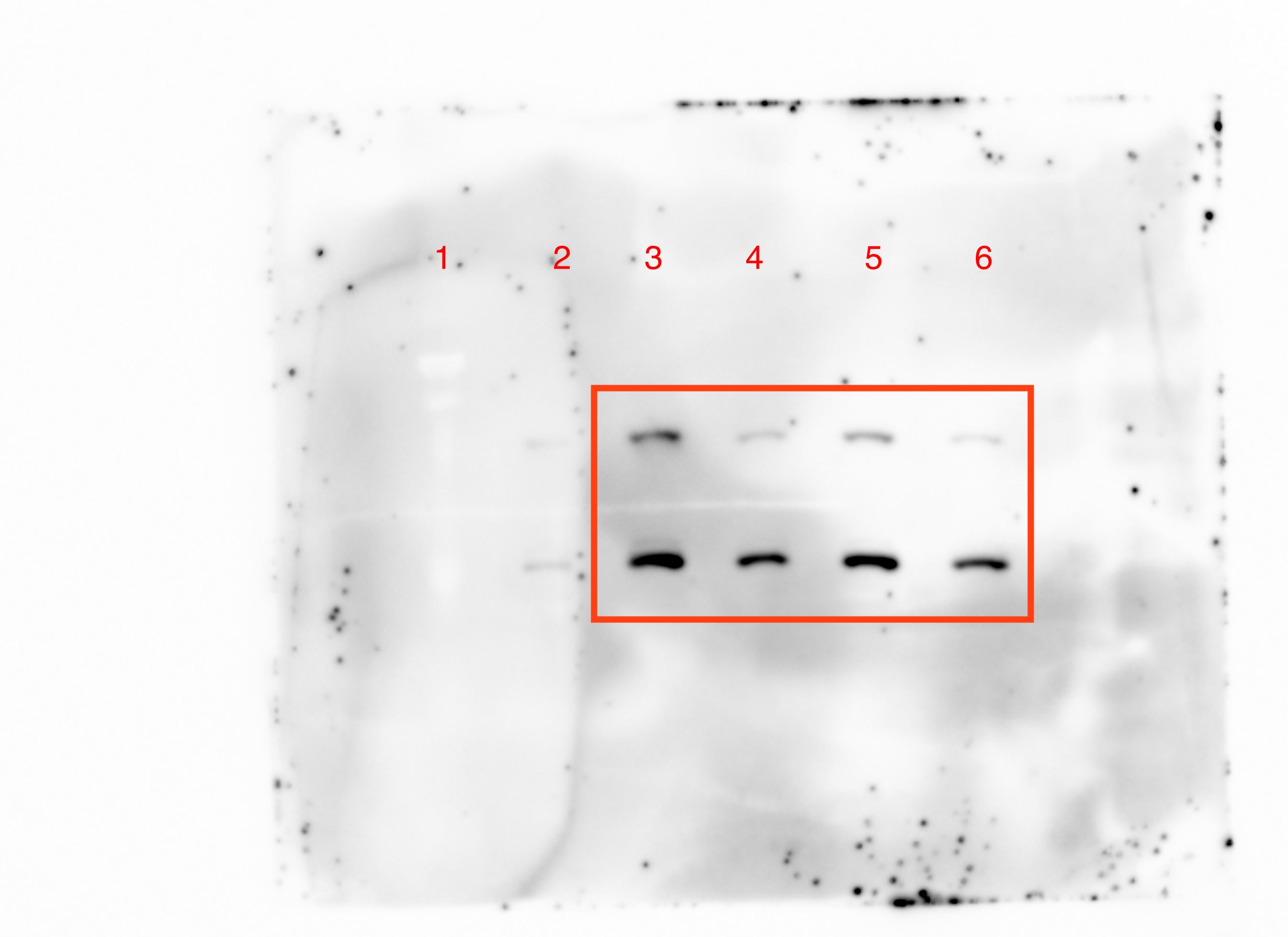

Supplement: Supplementary file 16 — Supplementary Information 16. [file 41598_2022_13525_MOESM16_ESM.tif]

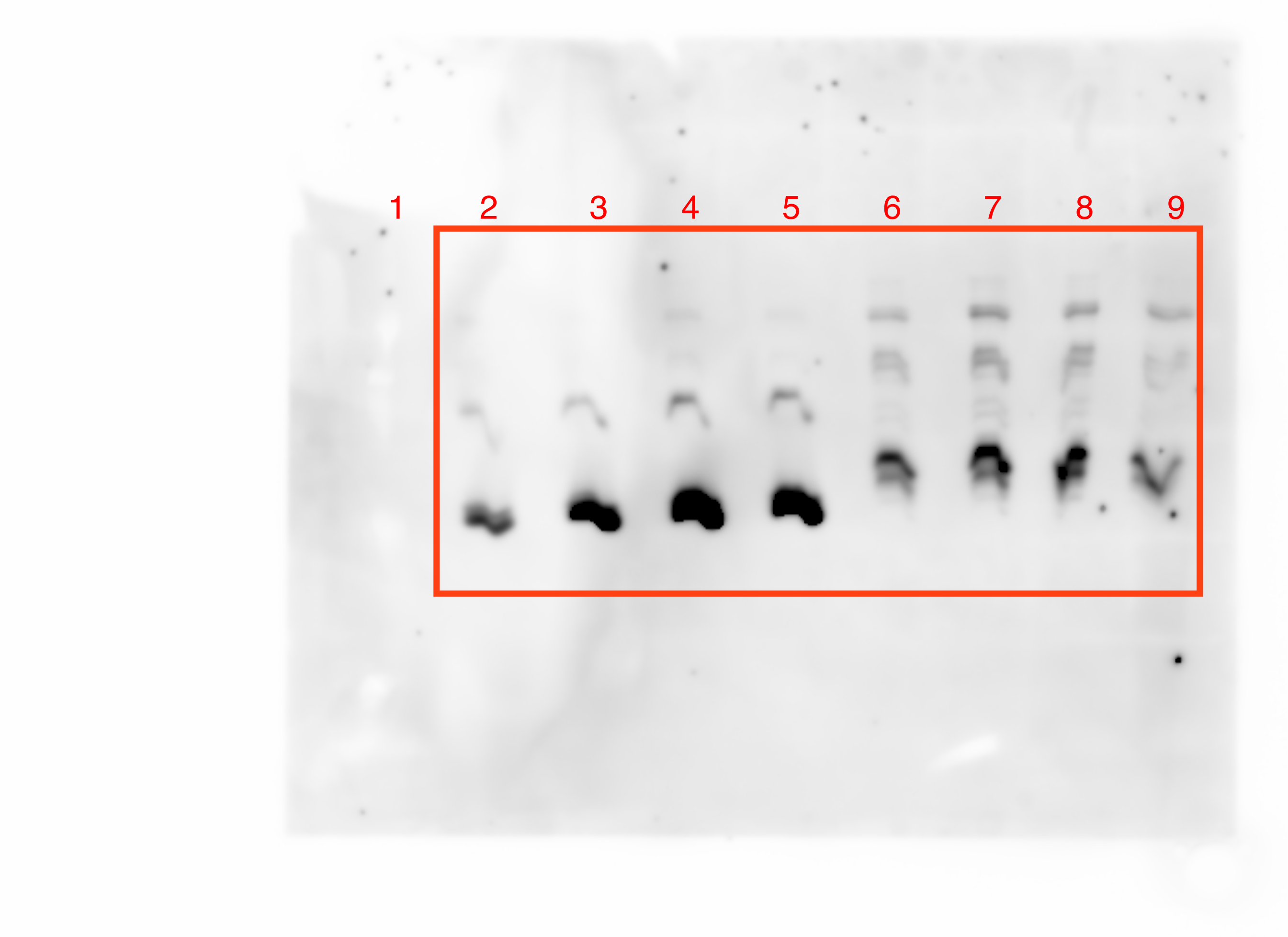

Supplement: Supplementary file 17 — Supplementary Information 17. [file 41598_2022_13525_MOESM17_ESM.tif]

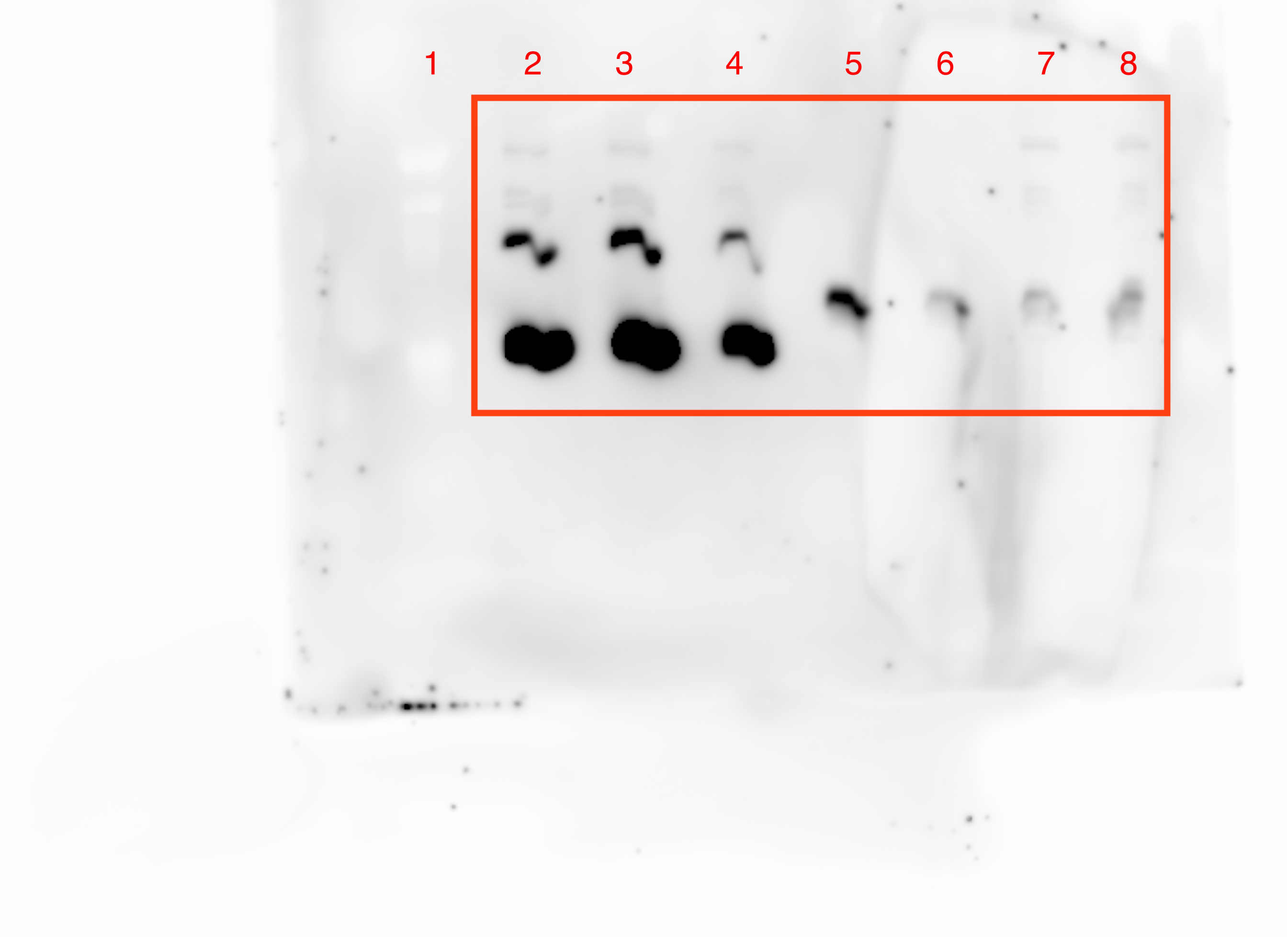

Supplement: Supplementary file 18 — Supplementary Information 18. [file 41598_2022_13525_MOESM18_ESM.tif]

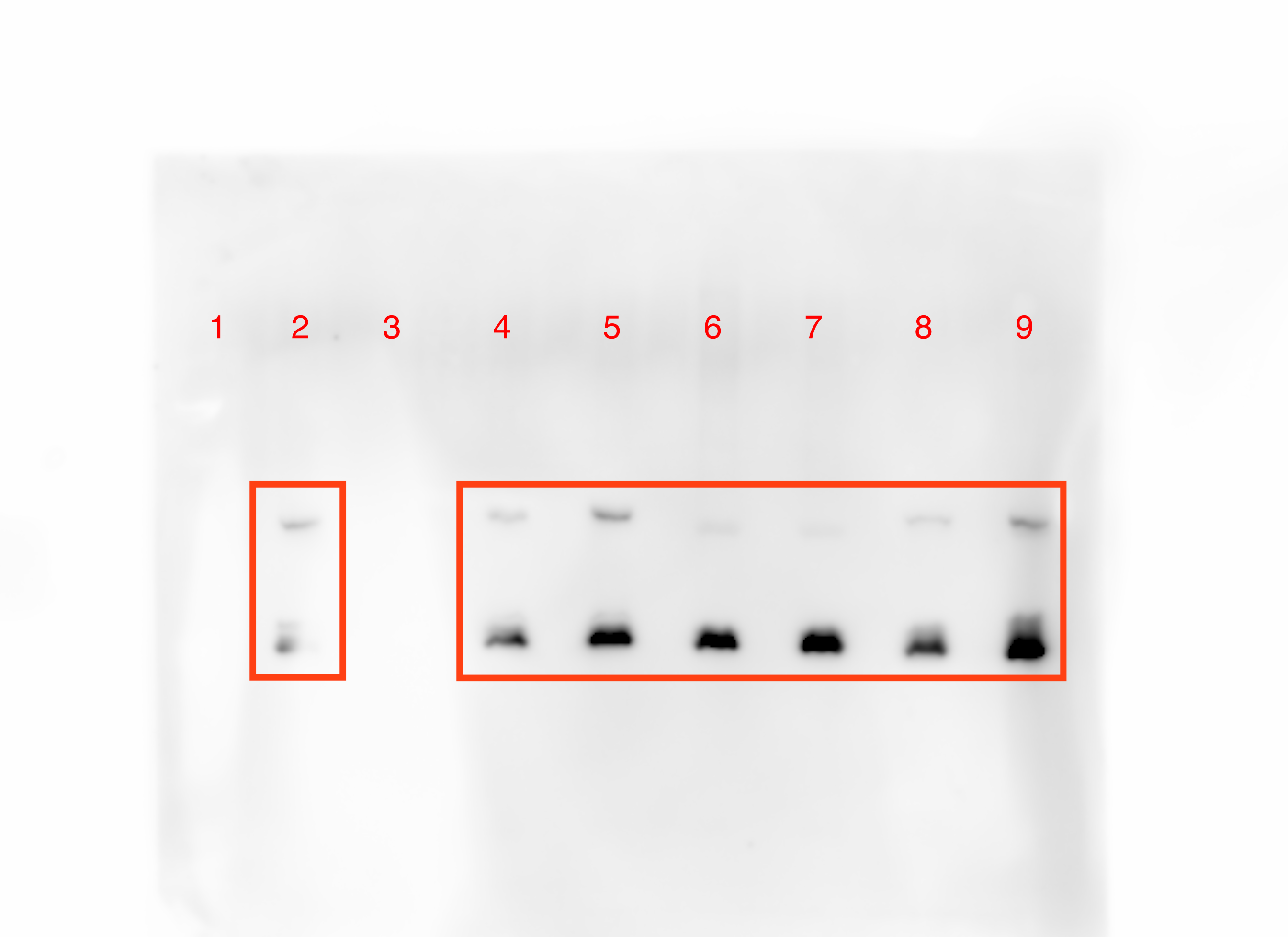

Supplement: Supplementary file 19 — Supplementary Information 19. [file 41598_2022_13525_MOESM19_ESM.tif]

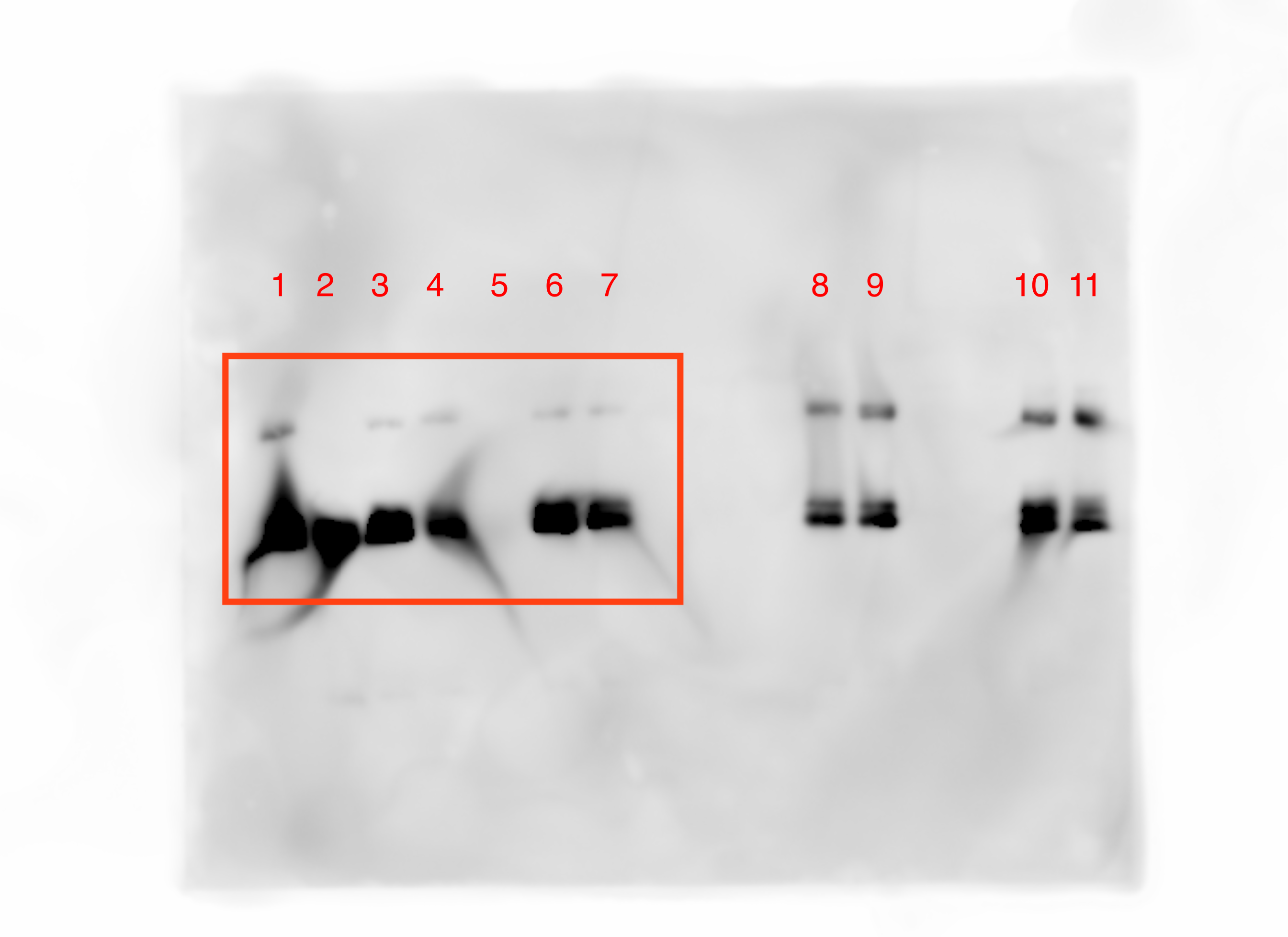

Supplement: Supplementary file 20 — Supplementary Information 20. [file 41598_2022_13525_MOESM20_ESM.tif]
